# Amino acid content in rice grains is affected by high temperature during the early grain-filling period

**DOI:** 10.1038/s41598-019-38883-2

**Published:** 2019-02-25

**Authors:** Min Huang, Hengdong Zhang, Chunrong Zhao, Guanghui Chen, Yingbin Zou

**Affiliations:** Sothern Regional Collaborative Innovation Center for Grain and Oil Crops (CICGO), Hunan Agricultural University, Changsha, 410128 China

## Abstract

Amino acid content in grains is an important nutritional quality trait in rice. High temperature can affect rice quality by accelerating grain filling. However, there is limited information available on the influence of high temperature on amino acid content in rice grains, especially under natural conditions. In this study, grain-filling traits and amino acid content in the grain of two rice cultivars (Luliangyou 996 and Lingliangyou 268) were compared between two years (2016 and 2017) with contrasting temperatures during the early grain-filling period under field conditions. Average daily mean temperature during the period of 0–5 days after full heading in 2016 (30.1 °C) was 5.4 °C higher than that in 2017. Initial, maximum, and mean grain-filling rates were 42–307% higher in 2016 than in 2017 for Luliangyou 996 and Lingliangyou 268. The time taken to reach the maximum grain-filling rate and active grain-filling duration were 6.3–10.7 d shorter in 2016 compared to 2017 for Luliangyou 996 and Lingliangyou 268. Grain weight was equal to or significantly higher in 2016 than in 2017 for Luliangyou 996 and Lingliangyou 268. N accumulation and N content in the grain were significantly lower in 2016 than in 2017 for both cultivars. The grain contents of all detected amino acids, except for methionine in Luliangyou 996, were significantly lower in 2016 than in 2017. Our study suggests that high temperature during the early grain-filling period can result in an accelerated grain-filling process, reduced N accumulation and content in rice grains, and consequently reduced amino acid content in the grain.

## Introduction

Rice is the staple food for more than half of the world’s population^1^. In China, rice production and consumption are the highest in the world, and China accounts for nearly one-third of the global rice market^2^. To meet the high demand for rice production due to the growing population and economic development, high yield has been the first priority of rice researchers in China for a long time^3^. In recent years, rice quality has been increasingly important to Chinese consumers as people’s living standards improve^4^.

Rice quality traits include physical appearance, cooking properties, sensory characteristics and, more recently, nutritional value^5^. Enhancing amino acids in the grain is one of the major objectives for improving the nutritional value of rice^6^. Basic research in genetics and genetic engineering research has led to useful information for enriching amino acid content in crops including rice^7^. However, rice quality depends not only on genetics but also on the environment^8^.

High temperature, especially during the grain-filling period, is one of the dominant environmental factors affecting rice quality^9^. It has been well documented that high temperature during grain filling can accelerate the grain-filling rate in rice, resulting in loosely packed rice starch granules, decreased head milled rice rate, and increased abnormal and chalky rice grains^10^. However, there is limited information available on the effect of high temperature during the grain-filling period on amino acid content in rice grains. Additionally, previous studies on high temperature affecting rice quality were mainly conducted under artificially controlled temperature conditions^10–12^, which may not fully reflect natural conditions.

In the present study, grain-filling traits and amino acid content in grains of two rice cultivars were compared between two years that differed due to a heat event during the early grain-filling period under field experiment conditions. The main objective of this study was to determine the influence of natural high temperature during the early grain-filling period on amino acid content in rice grains.

## Results

**Figure 1:**
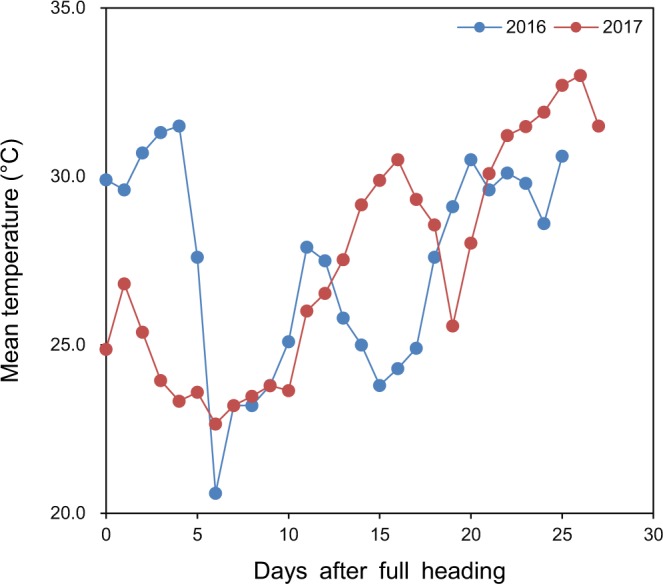
Daily mean temperature during the grain-filling period in the early rice-growing season in 2016 and 2017.

Average daily mean temperature during the period of 0–5 days after full heading in 2016 was 30.1 °C, which was 5.4 °C higher than that in 2017 (Fig. 1).

**Figure 2:**
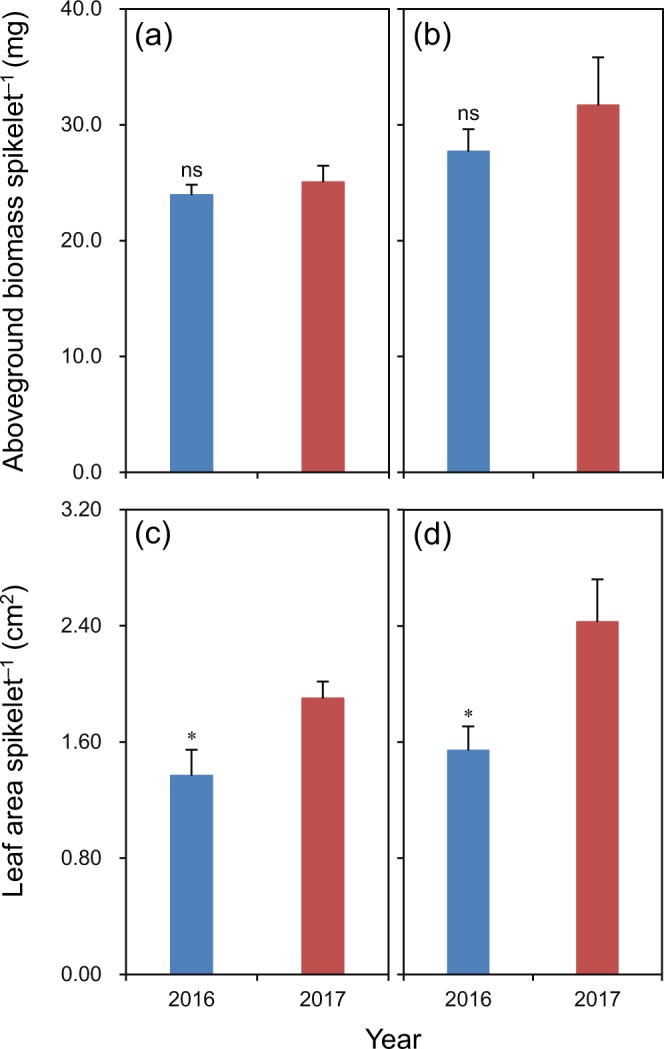
Aboveground biomass per spikelet (**a**,**b**) and leaf area per spikelet (**c**,**d**) at full heading of rice cultivars Luliangyou 996 (**a**,**c**) and Lingliangyou 268 (**b**,**d**) in 2016 and 2017. Error bars represent SE. ns and *denote non-significant and significant differences between the two years at the 0.05 probability level, respectively.

There was no significant difference in aboveground biomass per spikelet at full heading between 2016 and 2017 for Luliangyou 996 and Lingliangyou 268 (Fig. 2a,b). Leaf area per spikelet at full heading was 28% and 36% lower in 2016 than in 2017 for Luliangyou 996 and Lingliangyou 268, respectively (Fig. 2c,d).

**Figure 3:**
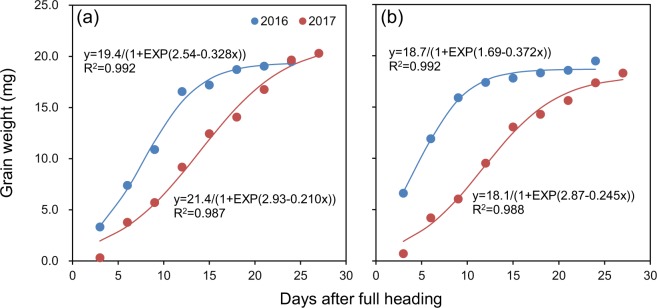
Grain-filling process fitted by logistic equation for rice cultivars Luliangyou 996 (**a**) and Lingliangyou 268 (**b**) in 2016 and 2017.

The grain-filling process was well fitted by the logistic equation (R^2^ = 0.987–0.992) for both cultivars in both years (Fig. 3a,b). The initial grain-filling rate was higher in 2016 than in 2017 by 100% for Luliangyou 996 and by 307% for Lingliangyou 268 (Table 1). Maximum grain-filling rate was 42% and 57% higher in 2016 than in 2017 for Luliangyou 996 and Lingliangyou 268, respectively. Mean grain-filling rate was higher in 2016 than in 2017 by 49% for Luliangyou 996 and by 86% for Lingliangyou 268. Time taken to reach the maximum grain-filling rate was 6.3 and 7.2 d shorter in 2016 than in 2017 for Luliangyou 996 and Lingliangyou 268, respectively. Active grain-filling duration was 10.7 d shorter in 2016 than in 2017 for both cultivars.

**Table 1:** Grain-filling parameters of two rice cultivars in 2016 and 2017.

Cultivar	Year	Grain-filling rate (mg grain^−1^ d^−1^)			Time taken to reach the maximum grain-filling rate (d)	Active grain-filling duration (d)
		Initial	Maximum	Mean		
Luliangyou 996	2016	0.431	1.591	0.892	7.7	15.9
	2017	0.216	1.124	0.597	14.0	26.6
Lingliangyou 268	2016	0.915	1.739	1.107	4.5	11.8
	2017	0.225	1.109	0.594	11.7	22.5

**Figure 4:**
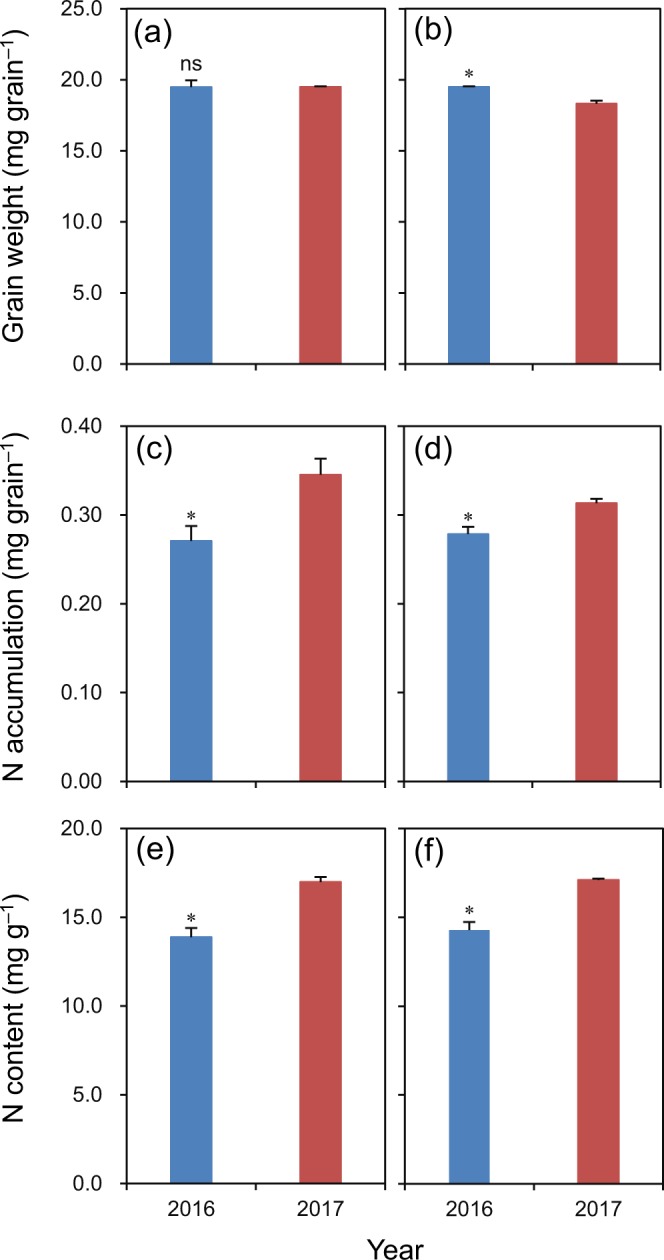
Grain weight (**a**,**b**), grain N accumulation (**c**,**d**), and grain N content (**e**,**f**) at maturity of rice cultivars Luliangyou 996 (**a**,**c**,**e**) and Lingliangyou 268 (**b**,**d**,**f**) in 2016 and 2017. Error bars represent SE. ns and *denote non-significant and significant differences between the two years at the 0.05 probability level, respectively.

The difference in grain weight was not significant between 2016 and 2017 for Luliangyou 996 (Fig. 4a). Grain weight was 6% higher in 2016 than in 2017 for Lingliangyou 268 (Fig. 4b). Grain N accumulation was 22% and 11% lower in 2016 than in 2017 for Luliangyou 996 and Lingliangyou 268, respectively (Fig. 4c,d). Grain N content was 18% and 16% lower in 2016 than in 2017 for Luliangyou 996 and Lingliangyou 268, respectively (Fig. 4e,f).

**Figure 5:**
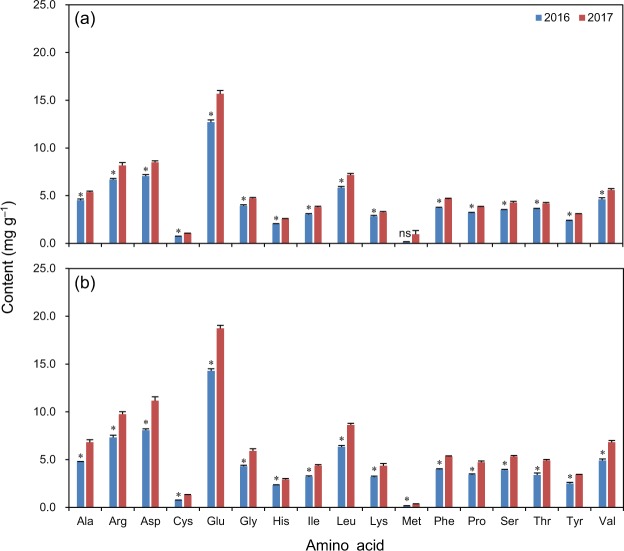
Amino acid content in grains at maturity of rice cultivars Luliangyou 996 (**a**) and Lingliangyou 268 (**b**) in 2016 and 2017. Ala, Arg, Asp, Cys, Glu, Gly, His, Ile, Leu, Lys, Met, Phe, Pro, Ser, Thr, Tyr, and Val represent alanine, arginine, aspartate, cysteine, glutamate, glycine, histidine, isoleucine, leucine, lysine, methionine, phenylalanine, proline, serine, threonine, tyrosine, and valine, respectively. Error bars represent SE. ns and *denote non-significant and significant differences between the two years at the 0.05 probability level, respectively.

For Luliangyou 996, the yearly differences were significant for the grain contents of all detected amino acids except methionine, which were 13–32% lower in 2016 than in 2017 (Fig. 5a). For Lingliangyou 268, there were significant yearly differences in the contents of all detected amino acids in grains, which were 20–49% lower in 2016 than in 2017 (Fig. 5b).

## Discussion

Most research studying the influence of high temperature during the grain-filling period on rice quality has been performed by artificially controlling temperature at a constant value during the whole period^10–12^. However, the natural temperature data presented in this study showed that the temperature was variable during the grain-filling period and the high temperature only occurred during a part of the grain-filling period (Fig. 1). This indicates that the approach of artificially controlling temperature at a constant value in previous studies is limited in that it does not necessarily reproduce natural conditions.

High temperature during the grain-filling period can accelerate the grain-filling process^13^. Consistently, in the present study, higher temperature during the early grain-filling period resulted in higher initial, maximum, and mean grain-filling rates and shorter time taken to reach the maximum grain-filling rate and active grain-filling duration in 2016 compared to 2017. Although the grain-filling rate can also be increased by increasing the source-sink ratio^14^, this was not responsible for the higher grain-filling rate during the early grain-filling period in 2016 compared to 2017 in this study because aboveground biomass and leaf area per spikelet at full heading were equal or smaller in 2016 than in 2017.

Accelerated grain-filling process is generally accompanied by enhanced remobilization of non-structural carbohydrate (NSC) from the vegetative tissues to the grain in rice, which can induce early plant senescence and reduce leaf photosynthesis^15^. However, these negative physiological changes do not necessarily result in reduced grain weight, because they can be compensated for or outweighed by gains from enhanced NSC remobilization and accelerated grain-filling rate^15,16^. This could also be why grain weight in 2016 was equal to or higher than that in 2017 in this study.

In contrast to the performance of grain weight, N accumulation in the grain, and thus grain N content, was lower in 2016 than in 2017. The decreased grain N accumulation in 2016 might be related to early plant senescence, which was induced by the accelerated grain-filling process. Consistently, Huang *et al*.^17^ observed that early plant senescence reduced N uptake during the grain-filling period and consequently decreased N accumulation in the panicle in rice plants. Tsukaguchi *et al*.^18^ found that protein content (calculated by multiplying N content by a factor of 5.95) in rice grains was most sensitive to plant nitrogen status after heading. However, there have been contradictory results showing that N uptake during grain filling is not always allocated to the grains and that rendering N remobilization from senescing organs acts as a central component for the development of reproductive organs in other cereal crops such as wheat^19^. Therefore, further research is required to quantify the sources of N accumulation in rice grains as affected by the early plant senescence induced by high temperature using the ^15^N tracer technique.

N is an important component of amino acids. Mossé *et al*.^20^ reported that amino acid contents increased linearly with N content in rice grains with correlation coefficients close to one. Therefore, in this study, the lower amino acid content in grains in 2016 compared to 2017 could be attributable to the decreased grain N content. On the other hand, the amino acid content can also be affected by changes in the activities of enzymes involved in amino acid metabolism in rice grains^21^. Glutamine synthetase, glutamic oxalo-acetic transaminase, and glutamate pyruvate transaminase, which play dominant roles in the conversion of inorganic N to organic N and the supply of effective N donors, are the key enzymes in amino acid synthesis in the rice grain. Therefore, further investigations are required to determine the effects of high temperature during the early grain-filling period on the activities of the above enzymes during grain development and their relationship with amino acid content in rice grains. Moreover, in contrast with the present study, Yamakawa and Hakata^22^ reported that high temperature resulted in amino acid accumulation in the rice grain due to the heat-stable import of amino acids into the caryopsis and/or repression of protein synthesis, especially during the tRNA charging step. This difference might be because the duration of high temperature was longer in the study of Yamakawa and Hakata^22^ (15 days) than in this study. High temperature led to decreased grain weight in the study of Yamakawa and Hakata^22^ but did not in the present study. These differences highlight that the need for greater fundamental understanding of the effects of the duration of high temperature on amino acid metabolism in the rice grain.

## Conclusion

High temperature during the early grain-filling period can result in accelerated grain-filling process, reduced N accumulation and content in rice grains and hence reduced amino acid content in the grain.

## Methods

Field experiments were conducted in Yongan (28°09′ N, 113°37′E, 43 m asl), Hunan Province, China in the early rice-growing season in 2016 and 2017. Normally, the temperature tends to increase with days after heading in the early rice-growing season^23^. This trend was observed in 2017 but not in 2016 in the present study (Fig. 1); in 2016, a heat event occurred during the early grain-filling period. Temperature data were obtained from an automatic weather station (Vantage Pro2, Davis Instruments Corp., Hayward, CA, USA) located near the experimental field.

The soil of the experimental field was clay with the following properties: pH 6.22, 41.8 g kg^−1^ organic matter, 1.23 g kg^−1^ total N, 0.71 g kg^−1^ total P, 6.56 g kg^−1^ total K, 132 mg kg^−1^ available N, 26.8 mg kg^−1^ available P, and 155 mg kg^−1^ available K. The soil test was based on samples taken from the 0−20 cm soil layer, with standard practices used for soil assessment.

In each year, Luliangyou 996, Lingliangyou 268, and other rice cultivars were planted in a randomized complete-block design with three replicates and a plot size of 40 m^2^. The other cultivars differed between the two years. To eliminate genetic factors, only Luliangyou 996 and Lingliangyou 268 were selected in this study. These two cultivars are hybrids and have been widely grown by rice farmers in the study region.

Seeds were sown in trays to raise seedlings. Twenty-day-old seedlings were transplanted with a high-speed rice transplanter (PZ80-25, Dongfeng Iseki Agricultural Machinery Co., Ltd., Xiangyang, China) on 15 April. Transplanting was done at a hill spacing of 25 cm × 11 cm with two seedlings per hill. Missing plants were manually replanted within 1 week after transplanting to obtain a uniform plant population. Nitrogen (150 kg N ha^−1^) was applied in three splits: 50% as basal fertilizer (1 day before transplanting), 20% at early-tillering (7 days after transplanting), and 30% at panicle initiation. Phosphorus (75 kg P_2_O_5_ ha^−1^) was applied as basal fertilizer. Potassium (150 kg K_2_O ha^−1^) was split equally at basal fertilization and panicle initiation. The experimental field was flooded (5–10 cm) from transplanting until 7 days before maturity, when plots were drained. Insects, diseases, and weeds were intensively controlled by chemicals.

Ten hills of rice plants were sampled from each plot at full heading (when more than 80% of plants showed panicles) and maturity. The plants sampled at full heading were separated into stems, leaves, and panicles. Leaf area was measured with a leaf area meter (LI-3000C, LI-COR, Lincoln, NE, USA). Dry weights of stems, leaves, and panicles were determined after oven drying at 70 °C to a constant weight. Aboveground biomass included the total dry matter of stems, leaves, and panicles. Plants sampled at maturity were hand threshed and filled spikelets were separated from unfilled spikelets by submerging them in tap water. The number of filled spikelets was counted using an automatic seed counter (SLY-A, Zhejiang Top Instrument Co., Ltd., Hangzhou, China). Aboveground biomass per spikelet was calculated by dividing the aboveground biomass at full heading by the number of filled spikelets. Leaf area per spikelet was calculated by dividing the leaf area at full heading by the number of filled spikelets.

About 80 main-stem panicles that headed on the same day were tagged in each plot. Six tagged panicles were sampled randomly from each plot, starting at 3 days after full heading at a 3-day interval until maturity. The grains were hand hulled and oven-dried at 70 °C to a constant weight. The grain-filling process was fitted using the logistic equation, and grain-filling parameters including initial, maximum and mean grain-filling rates, time taken to reach the maximum grain-filling rate, and active grain-filling duration were calculated according to Shi *et al*.^12^.

The dried grain samples at maturity were ground and sieved using a 100-mesh filter. About 0.5 g of the sieved grain sample was digested with H_2_SO_4_-H_2_O_2_, and the N content was determined using a segmented flow analyzer (Skalar SAN Plus, Skalar Inc., Breda, The Netherlands)^24^. Grain N accumulation was calculated by multiplying the grain weight by the N content.

Approximately 1.0 g of the sieved sample was hydrolyzed in HCl (5 M, 5 ml) for 22 h at 110 °C and derivatized using the AccQ-Tag reagent kit (Waters, Milford, MA, USA) for 15 min at 55 °C. The contents of amino acids, including alanine, arginine, aspartate, cysteine, glutamate, glycine, histidine, isoleucine, leucine, lysine, methionine, phenylalanine, proline, serine, threonine, tyrosine, and valine, were measured using a high-performance liquid chromatography system (Waters 2695 Separations Module, Waters) equipped with an AccQ-Tag column (3.9 × 150 mm, 4 μm film thickness) and linked simultaneously to a photodiode array detector (Model 2996, Waters)^25^.

Data were analyzed using analysis of variance (Statistix 8, Analytical software, Tallahassee, FL, USA). Means of years were compared based on the least significant difference test (LSD) at the 0.05 probability level for each cultivar.

## Data Availability

All data generated or analysed during this study are included in the article.
